# Clonal Expansion of Environmental Triazole Resistant *Aspergillus fumigatus* in Iran

**DOI:** 10.3390/jof6040199

**Published:** 2020-10-01

**Authors:** Fatemeh Ahangarkani, Hamid Badali, Kiana Abbasi, Mojtaba Nabili, Sadegh Khodavaisy, Theun de Groot, Jacques F. Meis

**Affiliations:** 1 Department of Medical Microbiology and Infectious Diseases, Canisius-Wilhelmina Hospital, 6532 SZ Nijmegen, The Netherlands; fkani63@gmail.com (F.A.); t.groot@cwz.nl (T.d.G.); 2 Antimicrobial Resistance Research Center, Communicable Diseases Institute, Mazandaran University of Medical Sciences, 4815733971 Sari, Iran; 3 Invasive Fungi Research Center, Communicable Diseases Institute, Mazandaran University of Medical Sciences, 4815733971 Sari, Iran; badalii@yahoo.com; 4 Fungus Testing Laboratory, Department of Pathology and Laboratory Medicine, University of Texas Health Science Center at San Antonio, San Antonio, TX 78229, USA; 5 Department of Microbiology, Zanjan Branch, Islamic Azad University, 4515658145 Zanjan, Iran; kiana.abbasi2000@gmail.com; 6 Department of Medical Sciences, Sari Branch, Islamic Azad University, 4815733971 Sari, Iran; m.nabili2010@gmail.com; 7 Department of Medical Parasitology and Mycology, School of Public Health, Tehran University of Medical Sciences, 1411734143 Tehran, Iran; sadegh_7392008@yahoo.com; 8 ECMM Excellence Center for Medical Mycology, Centre of Expertise in Mycology Radboudumc, Canisius-Wilhelmina Hospital, 6532 SZ Nijmegen, The Netherlands; 9 Bioprocess Engineering and Biotechnology Graduate Program, Federal University of Paraná, 80010 Curitiba, Paraná, Brazil

**Keywords:** *Aspergillus fumigatus*, azole resistance, compost, TR_34_/L98H, TR_46_/Y121F/T289A

## Abstract

Azole-resistance in *Aspergillus fumigatus* is a worldwide medical concern complicating the management of aspergillosis (IA). Herein, we report the clonal spread of environmental triazole resistant *A. fumigatus* isolates in Iran. In this study, 63 *A. fumigatus* isolates were collected from 300 compost samples plated on Sabouraud dextrose agar supplemented with itraconazole (ITR) and voriconazole (VOR). Forty-four isolates had the TR_34_/L98H mutation and three isolates a TR_46_/Y121F/T289A resistance mechanism, while two isolates harbored a M172V substitution in *cyp*51A. Fourteen azole resistant isolates had no mutations in *cyp*51A. We found that 41 out of 44 *A. fumigatus* strains with the TR_34_/L98H mutation, isolated from compost in 13 different Iranian cities, shared the same allele across all nine examined microsatellite loci. Clonal expansion of triazole resistant *A. fumigatus* in this study emphasizes the importance of establishing antifungal resistance surveillance studies to monitor clinical *Aspergillus* isolates in Iran, as well as screening for azole resistance in environmental *A. fumigatus* isolates.

## 1. Introduction

*Aspergillus fumigatus* is the most common agent of various forms of aspergillosis, including allergic bronchopulmonary aspergillosis (ABPA), chronic pulmonary aspergillosis (CPA), aspergilloma, and invasive aspergillosis (IA) [1]. Voriconazole (VOR) is the recommended primary and most effective therapy in the management of aspergillosis [2]. However, azole resistant *A. fumigatus* isolates are increasingly found worldwide with major epidemiological and clinical implications [3,4]. Therapeutic failure caused by azole-resistant *A. fumigatus* is becoming a significant concern to clinicians who are caring for patients at high risk for IA [1,4,5,6,7,8]. Azole resistance in *A. fumigatus* is mainly linked to *cyp51A*-mediated resistance mechanism, such as a 34-basepair (bp) sequence tandem repeat (TR_34_) in the promoter region of the *cyp51A* gene, in combination with a L98H substitution and a 46 bp tandem repeat (TR_46_) in the *cyp51A* promoter in combination with two amino acid changes (Y121F and T289A) in the CYP51A protein (TR_46_/Y121F/T289A) [9]. Isolates carrying these mutations exhibit a pan-azole resistant phenotype that can develop through long-term treatment with azole antifungals in the clinical setting or extensive exposure of the fungus to azole compounds in the environment [10,11]. Azole-resistant *A. fumigatus* with the TR_34_/L98H mutation isolated from environmental and clinical samples have been reported earlier in Iran, and we recently reported the occurrence of TR_46_/Y121F/T289A mutations in the *cyp51A* gene in *A. fumigatus* isolates from compost [12,13,14,15,16,17,18]. High concentrations of azole-resistant *A. fumigatus* spores are released during incomplete composting processes, especially when azole residues from agricultural waste are present [19]. Agricultural use of fungicides has driven the emergence and spread of azole-resistant *A. fumigatus.* The existence of an environmental route of azole resistance development involves serious risks for patients, as well, as they can become infected with azole resistant *A. fumigatus* strains before starting their treatment [12,13,14,15,16,17,18,19,20,21,22,23,24]. Notably, genetic exploration of azole resistant *A. fumigatus* strains indicates that isolates with the TR_34_/L98H allele are less genetically variable than susceptible isolates [12,23]. For instance, analysis of azole resistant *A. fumigatus* isolates in the Netherlands showed five distinct genotype groups in this country, while all the azole resistant isolates with the TR_34_/L98H mutation belonged to one group [5]. On the other hand, all clinical and environmental azole resistant *A. fumigatus* strains carrying TR_34_/L98H obtained from India were genetically identical [14]. These studies illustrate that *A. fumigatus* carrying this azole resistance mutation may preferentially spread clonal within a population. Major data gaps remain regarding the genotype distribution of azole resistance *A. fumigatus* in Iran. As ongoing reports indicate an expansion in the frequency of azole resistant *A. fumigatus* isolates worldwide, understanding the genetic structure of this potentially lethal fungus is critical. In this study, the genetic characterization of azole resistant *A. fumigatus* isolated from compost samples in Iran was explored.

## 2. Materials and Methods

### 2.1. Isolate Collection

According to a previously described protocol, commercial and home-made compost samples from different region of Iran (located about 300 km apart) were collected. To recover *A. fumigatus* strains, 1 cm^2^ of compost was dissolved in 5 mL sterile saline solution containing Tween 40 (0.05%), vortexed, and allowed to settle. For primary screening of azole-resistant *A. fumigatus* strains, 100 μL supernatant was plated on a Sabouraud dextrose agar plate (SDA; Difco, Franklin Lakes, NJ, USA), supplemented with 4 and 1 mg/L itraconazole and voriconazole, respectively, and incubated at 45 °C for 72 h in the dark [17]. Molecular identification of all *A. fumigatus* isolates that grew on the supplemented plate was performed with sequencing of the partial beta-tubulin gene as previously described [16].

### 2.2. In Vitro Antifungal Susceptibility Testing

Minimum inhibitory concentrations (MICs) were determined by broth microdilution susceptibility testing according to the methods in the Clinical and Laboratory Standards Institute (CLSI) reference standard (M38) [25]. For the preparation of the microdilution trays, itraconazole (Janssen, Beerse, Belgium) and voriconazole (Pfizer, Sandwich, UK) were obtained from the respective manufacturers as reagent-grade powders. All drugs were dissolved in 1% dimethyl sulfoxide (DMSO; Sigma, Zwijndrecht, the Netherlands) and were prepared at a final concentration of 0.031–16 mg/L. *Paecilomyces variotii* (ATCC 22319) and *Candida parapsilosis* (ATCC 22019) were used as quality controls [25].

### 2.3. Detection of Cyp51a Gene Mutations

All *A. fumigatus* isolates were subjected to a mixed-format real-time PCR assay specific for TR_34_/L98H and TR_46_/Y121F/T289A mutations of *cyp51A* gene leading to triazole resistance in *A. fumigatus* as described previously [26]. Those isolates with negative or inconclusive results in the real-time PCR assay, were further evaluated by sequencing the *cyp51A* gene as described previously [27].

### 2.4. Microsatellite Genotyping

Genotyping of all *A. fumigatus* isolates was performed with a panel of nine short tandem repeats (STRs) loci (namely short tandem repeats *Aspergillus fumigatus* (STR*Af*) 2A, 2B, 2C, 3A, 3B, 3C, 4A, 4B, and 4C), as previously described [28]. Genotypes were considered identical when they showed the same alleles for all nine loci [29,30]. Finally, the genetic relatedness between Iranian isolates from compost and 633 resistant *A. fumigatus* strains with clinical or environmental sources collected during 2001–2019 from different countries (The Netherlands, India, United Kingdom, Tanzania, France, Colombia, Romania, Ireland, China, Kuwait, Germany, and Japan) and previous Iranian isolates in the database at the Center of Expertise in Mycology, Radboudumc/Canisius-Wilhelmina Ziekenhuis (CWZ), in Nijmegen, The Netherlands, already barcoded using a panel of nine short tandem repeat loci, were analysed using BioNumerics software v7.6.1 (Applied Maths, Saint-Martens-Latem, Belgium).

## 3. Results

### 3.1. Triazole Resistant A. fumigatus with Mutation in cyp51A Gene

A total of 63 *A. fumigatus* colonies from 300 compost samples were obtained from SDA supplemented with itraconazole and voriconazole. Of these, 55 *A. fumigatus* isolates had high MICs of itraconazole (≥8 mg/L) and voriconazole (≥2 mg/L) by in vitro antifungal susceptibility testing. Exploring the mechanisms of resistance in these isolates by sequencing *cyp51A* and its promoter region showed that 44 isolates harbored the TR_34_/L98H mutation, three isolates the TR_46_/Y121F/T289A mutation and two isolates a M172V mutation. No mutations were found in 14 resistant isolates. Data of resistant isolates are summarized in Table 1. Details of isolates with the TR_46_/Y121F/T289A mutation have been previously described [17].

### 3.2. Microsatellite Typing Results and Evidence for Clonal Spread of a Single Triazole-Resistant A. fumigatus Genotype

**Figure 1:**
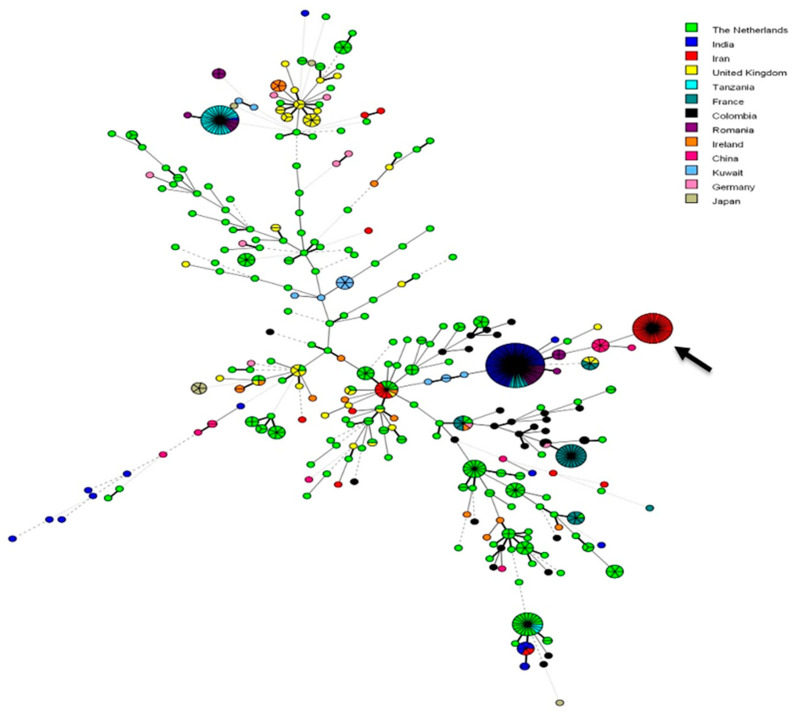
Minimum-spanning tree showing the genetic relationship of resistant *Aspergillus fumigatus* genotypes. Iranian clonal complex is illustrated by the arrow. Solid thick and thin branches demonstrate 1 or 2 microsatellite markers difference, respectively; dashed branches indicate 3 microsatellite markers difference between two genotypes; 4 or more microsatellite markers difference between genotypes are demonstrated with dotted branches.

Genotypic analysis identified that 41 *A. fumigatus* isolates with TR_34_/L98H shared the same allele across all nine examined microsatellite loci. These isolates came from compost in 13 different cities. The three remaining isolates with TR_34_/L98H exhibited three different genotypes. The two isolates with M172V differed by five microsatellite loci (2B, 2C, 3B, 3C, 4C). From the 14 azole resistant isolates with wild type *cyp51A*, which originated from 5 different cities, two isolates shared the same alleles across all nine microsatellite loci, while the 12 other isolates were genetically very diverse. A minimum spanning tree (MST) based on azole-resistant strains from various countries showed that the 41 Iranian *A. fumigatus* isolates with TR_34_/L98H formed a separate cluster (Figure 1).

## 4. Discussion

In this study, about 70% *A. fumigatus* isolates from compost samples grew on SDA supplemented with azoles and had the TR_34_/L98H mutation in the *cyp51A* gene. Indeed, the high rate of resistance to azole drugs due to the TR_34_/L98H mutation in *A. fumigatus* in Iran outperforms previous studies done during 2013–2016. The prevalence of clinical or environmental azole-resistant *A. fumigatus* isolates harboring this mutation was much lower in a previous episode and has been estimated between 3.2–6.6% [16,18,31]. Concurrent genetic studies of worldwide *A. fumigatus* isolates harboring the TR_34_/L98H resistance mechanism also suggested clonal expansion from a common resistant ancestor [32,33]. In the current study the azole resistant *A. fumigatus* population with TR_34_/L98H was grouped into four microsatellite genotypes, in which the genotype with STR*Af* profile: 2A:22, 2B:10, 2C:9, 3A:9, 3B:9, 3C:23, 4A:8, 4B:10, 4C:8 included 41 (93%) identical isolates, showing clonal expansion across different geographic locations. Furthermore, MST showed Iranian *A. fumigatus* isolates harboring TR_34_/L98H were apart from isolates of other countries and previously recovered Iranian isolates. Similar to our finding, Chowdhary et al. described a clonal spread and emergence of environmental azole resistant *A. fumigatus* strains carrying the TR_34_/L98H mutation from different parts of India. All Indian azole resistant isolates shared the same multilocus microsatellite genotype not found in any other analyzed samples within India or from other Asian or European countries [14]. In agreement with our findings, there is strong evidence that azole-susceptible or *cyp51A* single point mutation resistance strains have a greater genetic diversity than isolates harboring TR_34_/L98H and TR_46_/Y121F/T289A mutations, since the expansion of latter strains at a local level is predominantly clonal [14,34,35,36]. The dispersal of *A. fumigatus* with the TR_34_/L98H genotype supports the hypothesis that these strains have robust fitness in natural environments, with comparable or even higher fitness than that of wild-type strains [11]. Clonal spread of a single genotype in our study reinforced the hypothesis that geographic distances are not a barrier for the global spread from its centers of origin and their ability to cover thousands of miles by producing a large number of airborne spores or by anthropogenic means [14,31,37,38,39]. The widespread application of azole fungicides in Iran could have contributed to the spread of azole resistant *A. fumigatus* in environment niches, such as compost. To mitigate spread of azole resistant *A. fumigatus* in environment, changing of practices to prevent fungal diseases in plants on the fields is necessary. Procedures, such as prudent and restricted use of fungicides, controlling doses, and periods of fungicide application could be helpful. In cases where resistance to fungicides is observed, either the dosage can be increased or alternative fungicides can be used. In addition, environmental surveillance studies aimed to collect precise information of azole resistance monitoring to investigate the size and impact of this emerging problem is necessary [40].

Interestingly, we found that a sizable number of isolates (8 out of 54 resistant isolates) with azole MICs ≥16 mg/L exhibited no mutations in *cyp51A*. Other mechanisms of resistance, such as increased production of drug target Cyp51A protein, multidrug efflux pumps, or other proposed but not yet fully characterized mechanisms of resistance, such as amino acid substitutions in 3-hydroxy-3-methylglutaryl-CoA, stress response, and biofilm formation, can contribute to azole resistance in these isolates [32]. The limitation of our study was the absence of STR*Af* profiles of TR_34_/L98H *A. fumigatus* from neighbor countries of Iran, such as Pakistan or Turkey, for comparison with Iranian isolates [41,42]. In addition, the absence of clinical *A. fumigatus* was another drawback of our study. As most clinical microbiology laboratories in Iran do not routinely perform antifungal susceptibility testing of *Aspergillus*, the prevalence of azole resistance and mechanism of resistance in clinical *A. fumigatus* isolates in Iran is unknown [17].

## 5. Conclusions

Clonal spread of triazole resistant *A. fumigatus* isolated from compost, which is used widely in gardens and indoor plants in Iran, is concerning. This study highlights the importance of antifungal resistance surveillance studies of clinical and environmental *Aspergillus* isolates in Iran.

## Figures and Tables

**Table 1:** Description of all *A. fumigatus* isolates from compost.

Strain	Longitude and Latitude of Sampling	MIC (mg/L)			^3^ STR*Af*								
		^1^ ITR	^2^ VOR	Mutation in *cyp51A*	2A	2B	2C	3A	3B	3C	4A	4B	4C
mn224	35.9548° N, 52.1100° E	16	2	TR34/L98H	22	10	9	9	9	23	8	10	8
mn225	36.6717° N, 52.4439° E	16	2	TR34/L98H	22	10	9	9	9	23	8	10	8
mn226	36.6717° N, 52.4439° E	16	2	TR34/L98H	22	10	9	9	9	23	8	10	8
mn229	36.6717° N, 52.4439° E	16	2	TR34/L98H	22	10	9	9	9	23	8	10	8
mn227	36.7049° N, 52.6547° E	16	2	TR34/L98H	22	10	9	9	9	23	8	10	8
mn228	36.6329° N, 52.2667° E	16	2	TR34/L98H	22	10	9	9	9	23	8	10	8
mn231	36.4684° N, 52.8634° E	16	2	TR34/L98H	22	10	9	9	9	23	8	10	8
mn235	36.4684° N, 52.8634° E	16	2	TR34/L98H	22	10	9	9	9	23	8	10	8
mn232	36.4684° N, 52.8634° E	16	2	TR34/L98H	22	10	9	9	9	23	8	10	8
mn241	36.4684° N, 52.8634° E	16	2	TR34/L98H	22	10	9	9	9	23	8	10	8
mn233	36.6858° N, 52.5265° E	16	2	TR34/L98H	22	10	9	9	9	23	8	10	8
mn234	36.6858° N, 52.5265° E	16	2	TR34/L98H	22	10	9	9	9	23	8	10	8
mn236	36.4676° N, 52.3507° E	16	2	TR34/L98H	22	10	9	9	9	23	8	10	8
mn246	36.4676° N, 52.3507° E	16	2	TR34/L98H	22	10	9	9	9	23	8	10	8
mn247	36.5971° N, 52.6654° E	16	2	TR34/L98H	22	10	9	9	9	23	8	10	8
mn250	36.5659° N, 53.0586° E	16	2	TR34/L98H	22	10	9	9	9	23	8	10	8
mn251	36.5659° N, 53.0586° E	16	2	TR34/L98H	22	10	9	9	9	23	8	10	8
mn252	36.5659° N, 53.0586° E	16	2	TR34/L98H	22	10	9	9	9	23	8	10	8
mn253	36.5659° N, 53.0586° E	16	2	TR34/L98H	22	10	9	9	9	23	8	10	8
mn254	36.5659° N, 53.0586° E	16	2	TR34/L98H	22	10	9	9	9	23	8	10	8
mn255	36.5659° N, 53.0586° E	16	2	TR34/L98H	22	10	9	9	9	23	8	10	8
mn256	36.5659° N, 53.0586° E	16	2	TR34/L98H	22	10	9	9	9	23	8	10	8
mn257	36.5659° N, 53.0586° E	16	2	TR34/L98H	22	10	9	9	9	23	8	10	8
mn258	36.5659° N, 53.0586° E	16	2	TR34/L98H	22	10	9	9	9	23	8	10	8
mn260	36.5659° N, 53.0586° E	16	2	TR34/L98H	22	10	9	9	9	23	8	10	8
mn261	36.5659° N, 53.0586° E	16	2	TR34/L98H	22	10	9	9	9	23	8	10	8
mn263	36.5659° N, 53.0586° E	16	2	TR34/L98H	22	10	9	9	9	23	8	10	8
mn265	36.5659° N, 53.0586° E	16	2	TR34/L98H	22	10	9	9	9	23	8	10	8
mn266	36.5659° N, 53.0586° E	16	2	TR34/L98H	22	10	9	9	9	23	8	10	8
mn267	35.6892° N, 51.3890° E	16	2	TR34/L98H	22	10	9	9	9	23	8	10	8
mn268	35.6892° N, 51.3890° E	16	2	TR34/L98H	22	10	9	9	9	23	8	10	8
mn269	35.6892° N, 51.3890° E	16	2	TR34/L98H	22	10	9	9	9	23	8	10	8
mn270	35.6892° N, 51.3890° E	16	2	TR34/L98H	22	10	9	9	9	23	8	10	8
mn271	35.6892° N, 51.3890° E	16	2	TR34/L98H	22	10	9	9	9	23	8	10	8
mn272	35.6892° N, 51.3890° E	16	2	TR34/L98H	22	10	9	9	9	23	8	10	8
mn273	35.6892° N, 51.3890° E	16	2	TR34/L98H	22	10	9	9	9	23	8	10	8
mn274	35.6892° N, 51.3890° E	16	2	TR34/L98H	22	10	9	9	9	23	8	10	8
mn277	36.5659° N, 53.0586° E	16	2	TR34/L98H	22	10	9	9	9	23	8	10	8
mn279	36.9268° N, 50.6431° E	16	2	TR34/L98H	22	10	9	9	9	23	8	10	8
mn280	36.7284° N, 53.8102° E	16	2	TR34/L98H	22	10	9	9	9	23	8	10	8
mn281	36.7284° N, 53.8102° E	16	2	TR34/L98H	22	10	9	9	9	23	8	10	8
IFRC: 1854	35.6892° N, 51.3890° E	16	2	TR34/L98H	14	10	8	9	10	5	8	10	27
IFRC: 1858	35.6892° N, 51.3890° E	8	1	TR34/L98H	13	21	8	32	9	6	8	10	10
IFRC: 1866	35.6892° N, 51.3890° E	16	8	TR34/L98H	14	24	14	31	9	31	10	9	5
mn248	36.6329° N, 52.2667° E	16	16	M172V	11	15	16	19	29	4	7	5	8
IFRC: 1867	35.6892° N, 51.3890° E	16	16	M172V	11	16	9	19	20	5	7	5	5
IFRC: 1860	35.6892° N, 51.3890° E	16	0.125	Wild type	27	18	16	7	12	28	27	5	8
IFRC: 1868	35.6892° N, 51.3890° E	16	16	Wild type	27	18	16	7	12	28	27	5	8
IFRC: 1862	35.6892° N, 51.3890° E	16	0.5	Wild type	27	20	13	8	14	35	10	11	10
IFRC: 1864	35.6892° N, 51.3890° E	16	0.25	Wild type	20	10	8	37	9	6	10	9	15
IFRC: 1859	35.6892° N, 51.3890° E	16	0.25	Wild type	21	20	14	30	21	5	11	6	5
mn245	36.7049° N, 52.6547° E	16	1	Wild type	13	19	8	34	29	7	10	9	8
mn276	36.5659° N, 53.0586° E	16	2	Wild type	18	22	15	43	13	27	13	8	10
mn278	36.5659° N, 53.0586° E	16	2	Wild type	24	10	10	28	11	6	8	7	15
mn249	36.5659° N, 53.0586° E	0.125	0.5	Wild type	23	22	14	44	12	27	13	8	7
mn223	36.5659° N, 53.0586° E	0.125	0.5	Wild type	22	23	11	9	10	6	11	7	6
mn240	36.6329° N, 52.2667° E	0.5	1	Wild type	11	15	16	19	29	4	7	5	5
mn242	36.6329° N, 52.2667° E	0.125	0.5	Wild type	24	22	18	24	13	17	9	8	10
mn230	36.6717° N, 52.4439° E	0.5	0.5	Wild type	24	20	18	22	10	6	9	12	9
IFRC: 1863	35.6892° N, 51.3890° E	4	0.125	Wild type	24	18	15	94	10	6	14	11	10

^1^ ITR: itraconazole; ^2^ VOR: voriconazole; ^3^ STR*Af*: Short tandem repeats *Aspergillus fumigatus*.
